# A governance of ion selectivity based on the occupancy of the “beacon” in one- and four-domain calcium and sodium channels

**DOI:** 10.1080/19336950.2023.2191773

**Published:** 2023-04-19

**Authors:** J. David Spafford

**Affiliations:** Department of Biology, University of Waterloo, Waterloo, Ontario, Canada

**Keywords:** Calcium channels, sodium channels, ion selectivity, AlphaFold2 molecular modeling, patch clamp

## Abstract

One of nature’s exceptions was discovered when a Cav3 T-type channel was observed to switch phenotype from a calcium channel into a sodium channel by neutralizing an aspartate residue in the high field strength (HFS) +1 position within the ion selectivity filter. The HFS+1 site is dubbed a “*beacon*” for its location at the entryway just above the constricted, minimum radius of the HFS site’s electronegative ring. A classification is proposed based on the occupancy of the HFS+1 *“beacon”* which correlates with the calcium- or sodium-selectivity phenotype. If the *beacon* is a glycine, or neutral, non-glycine residue, then the cation channel is calcium-selective or sodium-permeable, respectively (Class I). Occupancy of a *beacon* aspartate are calcium-selective channels (Class II) or possessing a strong calcium block (Class III). A residue lacking in position of the sequence alignment for the *beacon* are sodium channels (Class IV). The extent to which animal channels are sodium-selective is dictated in the occupancy of the HFS site with a lysine residue (Class III/IV). Governance involving the *beacon* solves the quandary the HFS site as a basis for ion selectivity, where an electronegative ring of glutamates at the HFS site generates a sodium-selective channel in one-domain channels but generates a calcium-selective channel in four-domain channels. Discovery of a splice variant in an exceptional channel revealed nature’s exploits, highlighting the “beacon” as a principal determinant for calcium and sodium selectivity, encompassing known ion channels composed of one and four domains, from bacteria to animals.

## Introduction

### Identification of the family of animal voltage-gated sodium and calcium channels

Representatives of the eukaryotic voltage-gated sodium channels (Na_V_) were isolated by means of their high affinity to scorpion toxin [1] or saxitoxin [2] or tetrodotoxin (TTX) [3], which revealed a heavily glycosylated and large (~260 kDa) primary Na_V_α subunit first sequenced in electric organs of the electric eel [4] accompanied by smaller, accessory Na_V_β subunits first identified in mammals (~36 kDa) [5]. A high affinity to 1,4-dihydropyridines within rabbit T-tubules enabled purification [6] and sequencing [7] of a first eukaryotic voltage-gated calcium channel (Ca_V_), which like the Na_V_s, contains a large glycosylated pore-forming Ca_V_α_1_ subunit (175 kDa), in addition to an extracellular glycosylated Ca_V_α_2_ subunit Ca_V_δ (170 kDa), a cytoplasmic Ca_V_β subunit, and a unique muscle-specific Ca_V_γ transmembrane subunit (32 kDa). From these first representatives, the full complement of pore-forming subunits were identified, including nine sodium channels [8] Na_V_1.x (where x = 1 to 9) and seven high-voltage activated, calcium channels [9] Ca_V_1.1, Ca_V_1.2, Ca_V_1.3, Ca_V_1.4, Ca_V_2.1, Ca_V_2.2, and Ca_V_2.3 in mammals. Later, a class of three low-voltage activated T-type calcium channels were identified in mammals [9], including Ca_V_3.1, Ca_V_3.2 and Ca_V_3.3, by means of their sequence homology to Ca_V_1.x, Ca_V_2.x calcium channels [10]. Na Leak ChaNnel, “NALCN,” was also identified in mammals also by means of sequence homology [11] and is distributed in animal genomes usually as a single gene (outside of two genes in some species of sponges, cnidarians and nematodes) and is a distant relative resembling both eukaryotic Ca_V_ and Na_V_ channels [12].

## Calcium channel structures are conserved from choanoflagellates (non-animals) to humans

**Figure 1.**
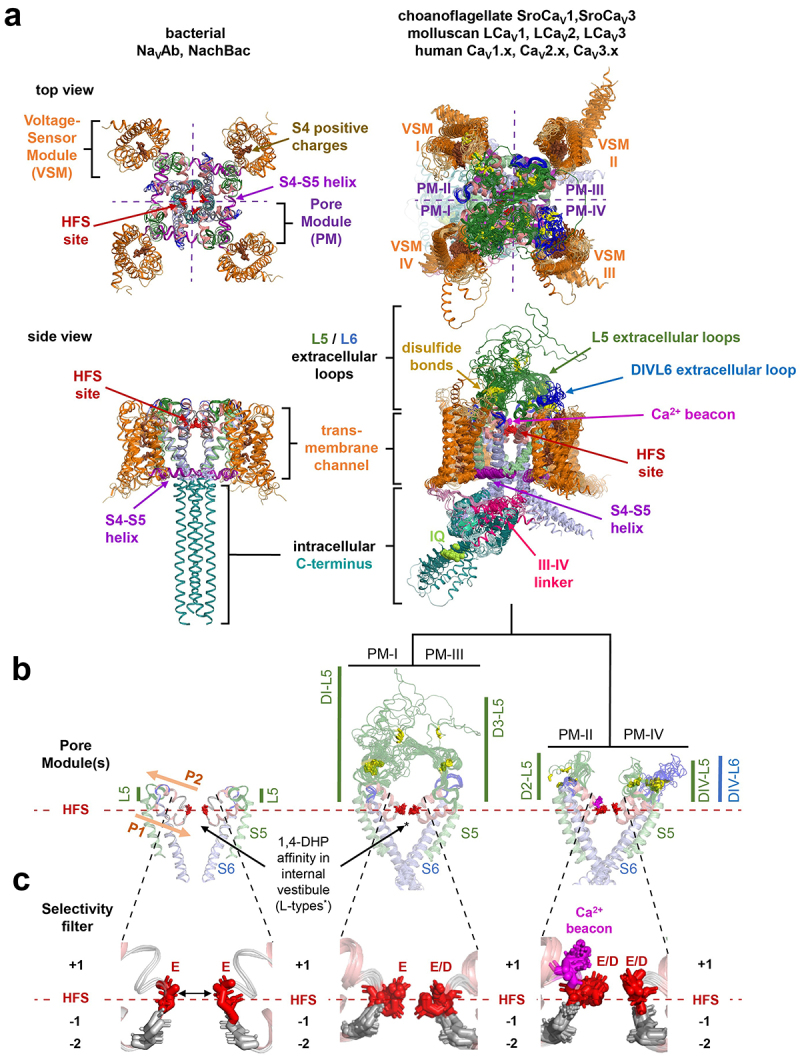
A “*beacon*” aspartate residue resides in the (High Field Strength) HFS^+1^ position of Domain II of the outer pore in choanoflagellate and animal calcium-selective Ca_V_1, Ca_V_2 and Ca_V_3 channels. Overlay of experimentally derived prokaryotic sodium channel structures by X-ray crystallography and eukaryotic calcium channels by single nanoparticle cryo-EM (identified by PDB identification number). Indicated ion channels in Figure 1 lacking experimental structures were computationally derived AlphaFold2 models. Structures illustrated in PyMOL 2.5.2 (Schrödinger, LLC) include bacterial channels: NavAb (Arcobacter butzleri), PDB ID: 5vb2 [13], and NaChBac (*Alkalihalobacillus halodurans* C-125) PDB ID: 6vx3 [14;] Choanoflagellate (*Salpingoeca rosetta*) channels: SroCa_V_1, SroCa_V_3; Molluscan (*Lymnaea stagnalis*) channels: LCa_V_1 [15], LCa_V_2 [16] and LCa_V_3 [17;] human channels (except Ca_V_1.1 which is rabbit): Ca_V_1.1 (PDB ID: 6jp8), Ca_V_1.2, Ca_V_1.3 (PDB ID: 7uhg), Ca_V_1.4, Ca_V_2.1, Ca_V_2.2 (PDB ID: 7vfs), Ca_V_2.3, Ca_V_3.1 (PDB ID: 6kzp), Ca_V_3.2, Ca_V_3.3 (PDB: 7wli). Illustrated in Panels: (A) Top and side views; (B) Pore module only view; (C) Closeup of HFS^−2^ to HFS^+1^ positions of the selectivity filter. Eukaryotic channels dramatically vary from homo-multimeric bacterial channels in the asymmetric pore modules of four domains with large L5/L6 extracellular loops forming a windowed dome over the pore, populated with cysteine-bridges (yellow color). The selectivity filter is formed by the high field strength site (HFS) of negatively charged residues (D, E), two rings of backbone carbonyls (−1/-2 position), a *beacon* aspartate (in Domain II HSF+1 position) of Ca^2+^ selective channels, and negatively charged residues forming an outer ring (+3 or +4 position).

Overlay of fifteen experimentally (single nano-particle cryo-EM) and computationally derived (AlphaFold2) structures illustrates a high degree of structural conservation between representative calcium channels (Figure 1). These overlayed structures in Figure 1 include the simplest known eukaryotes to contain animal homologs to Ca_V_α_1_ subunits: the L-type (SroCa_V_1) and T-type (SroCa_V_3) channel from the Clade I Craspedida choanoflagellate, *Salpingoeca rosetta*. Representative invertebrate homologs overlaid in Figure 1 include LCa_V_1 [15], LCa_V_2 [16] and LCa_V_3 [17] from the mollusk, *Lymnaea stagnalis* and the ten known human channels, Ca_V_1.x (x=1 to 4), Ca_V_2.x (x=1 to 3) and Ca_V_3.x (x=1 to 3) [9]. Eukaryotic Na_V_ and NALCN channels overlap in general assembly of the pore-forming subunit as the Ca_V_ channels overlaid in Figure 1. These voltage-gated cation channels consist of four homologous domains (designated DI−DIV), each containing a peripheral voltage-sensor module (VSM) at the corners of a pore-module (Figure 1). The mobile alpha helix of S4 segments contains voltage-sensing positive charges every third residue and counter-charges in S1, S2 and S3 segments [18] (Figure 1). The S4-S5 amphipathic helices serve as lever arms which couple the VSM movements to opening and closing of the central channel orifice created by an inverted “teepee”-like bundle crossing of S6 segments of the central pore module (PM) [19]. The long S4-S5 helices position the VSM rotating turned with respect to PM of each domain (Figure 1). This domain swapping arrangement supports a rotary coupling of interlocked PMs for more concerted actions in gated pore movements [20]. This domain swapping arrangement is absent in many voltage-gated cation channels (including EAG [21], CNG [22], HCN [23], KCa [24], KNa [25], and some TRP channels [26], because their S4-S5 helices are too short.

## A lysine residue in Domain II or Domain III of the high field strength (HFS) site is a major determinant to the sodium selectivity of animal Na_v_1 and NALCN channels

**Figure 2.**
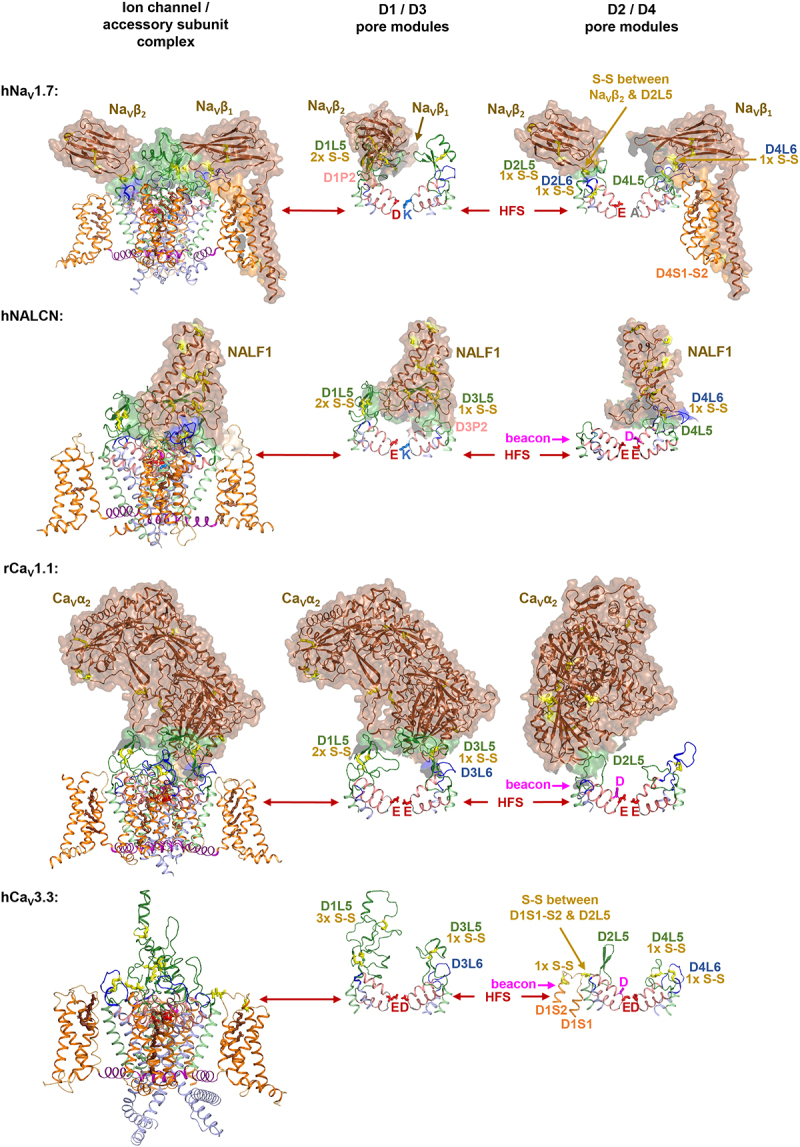
Extracellular loop regions are saddled with bulky accessory subunits on Na_V_1, Ca_V_1/Ca_V_2 and NALCN channels, but not on Ca_V_3 channels. Cryo-EM structures of mammalian channels: Human Na_V_1.7, PDB ID: 6j8i [30;] rabbit Ca_V_1.1, PDB ID: 6jpa [31;] human Ca_V_3.3, PDB ID [32], and human NALCN, PDB ID: 7s×4 [33]. Arrangements of extracellular loops (L5=green color, L6=blue color) and accessory subunits (brown color) are shown with 50% transparency. Key determinants for ion selectivity include the *beacon* (HFS^+1^) and the high field strength (HFS) site are shown in the D1/D3 and D2/D4 panels.

The pore module (PM) contains a membrane reentrant pore loop (P-loop) between an outer S5 helix and inner S6 helix, leaving an inverted-prism shaped aqueous vestibule within the channel pore delimited by the selectivity filter and the flanking “V” shapes contributed by the S5/S6 helices (Figure 1). The aqueous vestibule in the pore between the selectivity filter above and the channel gates below is contoured for the high affinity occupancy of state-dependent ligands, such as the 1,4 dihydropyridines, the phenylalkylamines, and benzothiazepines in the L-type calcium channels [27] (Figure 1). A variable cysteine-enriched loop L5 rises into the extracellular space from the top of the outer S5 helix, and then descends from the top of the membrane by means of a P1 pore helix to a constriction point for ion passage of pore-lining residues contributing to the ion selectivity filter (SF) in the outer pore region. The L5 loop continues as the membrane-ascending P2 pore helix to a variable, cysteine-enriched L6 extracellular region looping above the membrane before connecting to the apex of the S6 helix (Figure 1). Voltage-gated K_V_ channels differ in lacking the P2 pore helix [28], likely because of the greater necessity of secondary structure to stabilize the asymmetrical pores of the Na_V_ and Ca_V_ channels [29], especially since these four-domain channels are usually burdened with extracellular accessory subunits (see Figure 2). The selectivity filter (SF) consists of neutral (inner and central) residues contributing carbonyls forming the inner causeway for ion passage to the internal aqueous vestibule below and side chains of negatively charged residues that form the high field strength (HFS) site above (Figure 1) [34]. There are often side chains of negatively charged residues in the HFS+3 and HFS+4 position in the outer rim of the pore selectivity filter, that serve to attract cations from the extracellular space by don’t directly determine a pore’s ion selectivity [35], in a manner similar to ClC, voltage-dependent chloride channels which possess positively charged residues in the pore’s outer rim to attract anions [36]. The side chain residues that make up the HFS site in DI, DII, DIII and DIV are EEEE or EDEE in Ca_V_1 and Ca_V_2 channels or EEDD in Ca_V_3 channels (Figure 1). The ubiquity of a HFS composed only of negatively charged residues in Ca_V_ channels is broken in animal Na_V_1 channels with a lysine residue in Domain III: DEKA (most animal Na_V_1 channels) [37,38], or Domain II: DKEA (cnidarians only) [39], and placement of a neutral residue in Domain IV (A or G) (Table 1). Replacing negative charges (D or E) in the HFS site for the positively charged lysine in Domain II or III demonstrates a transference of calcium to sodium selectivity, and vice versa [37,38]. A governance of sodium selectivity determined by a lysine in Domain III (DEKA) or Domain II (DKEA), is supported in the lack of preference of passage for sodium or calcium ions in eukaryotic Na_V_2 channels which possess a HFS site always lacking a lysine residue in Domain III or II (DEEA) [40] (Table 1). The nonselective Na_V_2 channels are an ancestral lineage of sodium channel found in species within the Obazoa [41], which includes apusozoan and choanoflagellate species of single cell eukaryotes, and animals but notably lacking in bony vertebrates [42]. NALCN channels possess a consistency in sodium-like pores with a lysine residue of the HFS site in Domain III: EEKE (nematodes, arthropods, vertebrates), or Domain II: EKEE (annelids, mollusks, echinoderms, hemichordates) [12] (**see** Table 1). Animal phyla containing species primarily inhabiting the marine, not terrestrial or freshwater environment, possess an alternative or exclusive calcium-like, EEEE HFS site within their NALCN channel, resembling eukaryotic Ca_V_ channels, and lacking a lysine residue in Domains III or II [12] (Table 1).

**Table 1.** Distribution of cysteine bridges in extracellular loops and outline of key determinants for calcium and sodium permeation through calcium, sodium and NALCN channels composed of one- and four-domains channels in bacteria and eukaryotes.

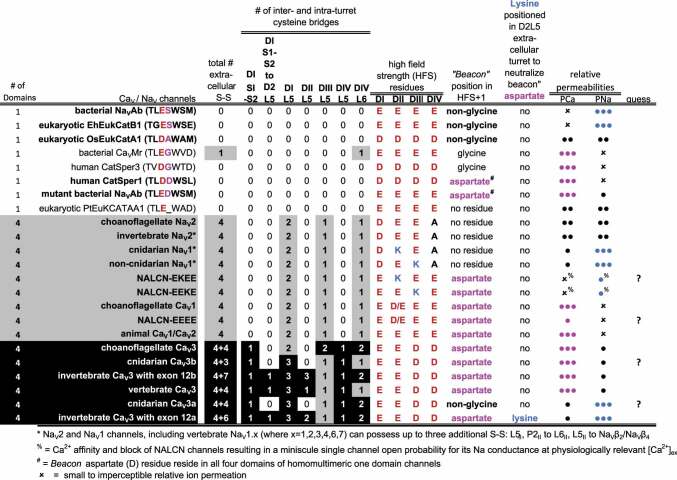

## Selective pores of prokaryotic sodium channels resemble eukaryotic calcium channels, not animal Na_V_1 sodium channels

The NaChBac channel isolated from *Bacillus halodurans* was the first Na_V_ channel characterized from bacteria [43] and its close relative NavAb from *Arcobacter butzleri* was the first to be resolved at atomic resolution by X-ray crystallography [34]. Prokaryotic Na_V_ channels comprise a tetramer of four subunits (Figure 1) [34], much like the prokaryotic [28] and eukaryotic [44] voltage-gated potassium channels (K_V_s), with each subunit corresponding to one of the four domains of the animal Na_V_ [4] and Ca_V_ [7] channels. These prokaryotic Na_V_ channels are homo-tetramers with a single HFS site containing a ring of four glutamate residues (EEEE) resembling animal Ca_V_ channels [34,45], but not resembling animal Na_V_1 channels with the lysine residue in a HFS site (Figure 1). Resemblances of bacterial Na_V_ channels to animal Ca_V_ channels extends to pharmacological blockade by low micromolar affinity to 1,4-dihydropyridines, like the L-type, Cav1 channels, but not to Nav1 channel pore blocker, tetrodotoxin (TTX) [43]. High affinity binding of 1,4-dihydropyridines likely reflects the similarity of the prism-shaped internal vestibule of bacterial Na_V_ channels and animal Cav1 channels (Figure 1) and ligand interaction with residues in S5_III_, S6_III_, S6_IV_ and P1_III_ [27,46].

Bacterial Na_V_Ab channels can be artificially converted into calcium-selective channels after replacement of three aspartates in the selectivity filter (TL**E**SWSM to TL**D**DWSD), which translates into 12 amino acid changes to the homotetrameric Na_V_ channel [47]. On the other hand, if an aspartate (D) replaces serine (S) at the HFS^+1^ position only (immediately C-terminal to the glutamate) at the HFS site, there is a dramatic 100-fold increase in permeability of calcium over sodium ions in bacterial Na_V_ channels, TL**E**SWSM to TL**E**DWSM [47] (Table 1).

A single serine-to-aspartate mutation at the HFS^+1^ site of the one-domain, homo-tetrameric bacterial Na_V_ channels is translated to all the four subunits. An aspartate in just one of four HFS^+1^ sites (Domains II or Domains IV) is ubiquitously featured in four domain, calcium-selective Ca_V_1, Ca_V_2 and Ca_V_3 channels of eukaryotes (Figure 1 and Table 1). The nomenclature “*beacon*” is dubbed to the ubiquitous HFS^+1^ aspartate in Domain II following the negatively charged HFS. Replacement of the *beacon* with electroneutral asparagine (N) renders sodium-permeability to animal Ca_V_ channels [48,49]. And in the opposite scenario, if the “*beacon*” aspartate is engineered in bacterial Na_V_ channels, they become calcium-selective [47].

## NALCN channels appear as hybrids between animal calcium and sodium channels

NALCN channels appear to have arisen as hybrids distantly resembling both the animal Na_V_ and Ca_V_ channels from which they likely originated in single-cell eukaryotes. NALCN channels possess a “*beacon*” aspartate in HFS^+1^ positioned in Domain IV instead of Domain II of the animal calcium-selective Ca_V_1, Ca_V_2 and Ca_V_3 channels (Table 1) [50,51]. NALCN channels in different animal species can possess exclusively or alternatively, a Na_V_1 channel-like HFS site containing a lysine (K) in Domain II or Domain III [12] (Table 1), which is critical for its preference of a sodium leak over calcium leak conductance [52]. Human NALCN possesses an extremely low single-channel open probability (0.04 ± 0.01), providing NALCN with a mostly non-conducting phenotype even during periods of high activity. A near-complete Ca^2+^ block of the Na^+^ conductance [33] may be contributed in part by the beacon aspartate in Domain IV. Presence of the beacon aspartate in Domain IV instead of Domain II of animal Ca_V_1, Ca_V_2 and Ca_V_3 channels ensures that positioning of the calcium beacon does not interfere with the lysine residue, which can reside in the HFS site of NALCN in Domain II. The small open probability of NALCN and the massive, intertwined, HEAT-repeats in UNC-79 and UNC-80 intracellular subunits [33] suggest that NALCN may also serve as a regulatory sensor contributing to electromechanical coupling triggering intracellular cascades in addition to providing a possible ion channel conductance when external Ca^2+^ is very low or absent [53].

## Greater variability in L5/L6 extracellular loops of Ca_V_3 channels in response to a lack of an extracellular subunit capping Ca_V_3 channels

In analyzing a novel splice variant, “exon 12a” spanning the extracellular L5 loop in Domain II of a molluscan Ca_V_3 T-type channel, LCav3, from the giant pond snail, *Lymnaea stagnalis*¸ unusually large outward currents carried by 100 mM internal Cs^+ 49^ were observed. Cs^+^ ions are retained at high concentrations in the patch pipette during whole-cell patch clamp recording to prevent monovalent ion currents through potassium channels [54]. This led to realization of a likely novel structural feature without precedence, of a short extracellular L5 loop designed to dramatically increase the relative monovalent ion (sodium) permeability through a Ca_V_3 T-type calcium channel without mutation of the HFS site or other selectivity filter residues. More than twenty mutations and chimeric Ca_V_3 channels were analyzed to elucidate a potential mechanism [48,49]. An important question is why switching a preference in passage of calcium to sodium ions is limited to animals in two invertebrate phyla, and is not present outside of Ca_V_3 channels? A first clue of what makes Ca_V_3 channels unique is illustrated in Figure 2.

As illustrated, Na_V_, Ca_V_, and NALCN channels in animals, but not prokaryotes possess a framework of extracellular loops which are engaged in supporting different extracellular subunits of very different sizes and shapes. The subunits could be imagined as a “*tipped over party hat*” (NALF1/2 on top of NALCN) [50,51], “*Mickey Mouse ears held up by a string*” (Na_V_β subunits on top of Na_V_1.x channels of the bony vertebrates) [55], and “*a sitting puppy with an oversize head*” (Ca_V_α_2_ subunit on top of Ca_V_1 and Ca_V_2 channels) [56] (Figure 2). The extracellular subunits are variable, but the framework of extracellular loops on which the extracellular subunits are saddled upon are overlapping in these ion channels. Lack of constraints on the structure of extracellular loops are evident within the Ca_V_-like channels outside the animals, which often lack homologs to animal Ca_V_α_2_ subunit, and within the Ca_V_3 channels which lack any obligate accessory subunits. These channels without obligatory extracellular subunits can dramatically vary in their dimensions and cysteine bridge content in extracellular loops compared to Ca_V_1, Ca_V_2, Na_V_, and NALCN channels in animals. A particular asymmetry of large extracellular loops is a significant and a shared feature of the eukaryotic four-domain channels (Figure 1). The largest of the extracellular loop in Na_V_, Ca_V_1/2/3 and NALCN channels is always L5_I_ (60 to 110 amino acids), followed by L5_III_ (40 to 65 amino acids), with the longest L6 loop being in Domain IV (L6_IV_). Which of these large extracellular loops is engaged in binding extracellular subunits varies, with a lack of engagement of Na_V_β subunits on L5/L6_III_, NALF1 on L5/L6_II_, or Ca_V_α_2_ on L5/L6_IV_ (Figure 2). The shortest of the extracellular loops lie across from one another in the pore of L5/L6_II_ and L5/L6_IV_ (15 to 30 amino acids) very close to the membrane, compared to longer L5/L6_I_ and L5/L6_III_ extracellular loops (Figure 1, side view) which loom high above and partially enclose the selectivity filter below like a windowed dome (Figure 1, top view).

There is a consistency in four intra-turret cysteine bridges populating Na_V_, Ca_V_1/2 and NALCN channels with two bridges in L5_I_, one bridge in L5_III_ and one bridge in L6_IV_ (Figure 1, Table 1). Notable exceptions to the four intra-turret disulfides rule are in many but not all Na_V_2 and Na_V_1 channels, which can possess up to three additional disulfides spanning L5_III_, P2_II_ to L6_II_ and L5_II_ to Na_V_β_2_ or Na_V_β_4_ [5,55,57]. The optional presence of these three disulfides in Na_V_ channels appears to correlate with the variability in Na_V_β subunit structures, which are not homologous within different animal phyla [58].

## Calcium channels can possess extra cysteine bridges in L5 and L6 extracellular loops, which can influence the pore’s calcium selectivity and sodium permeability

**Figure 3.**
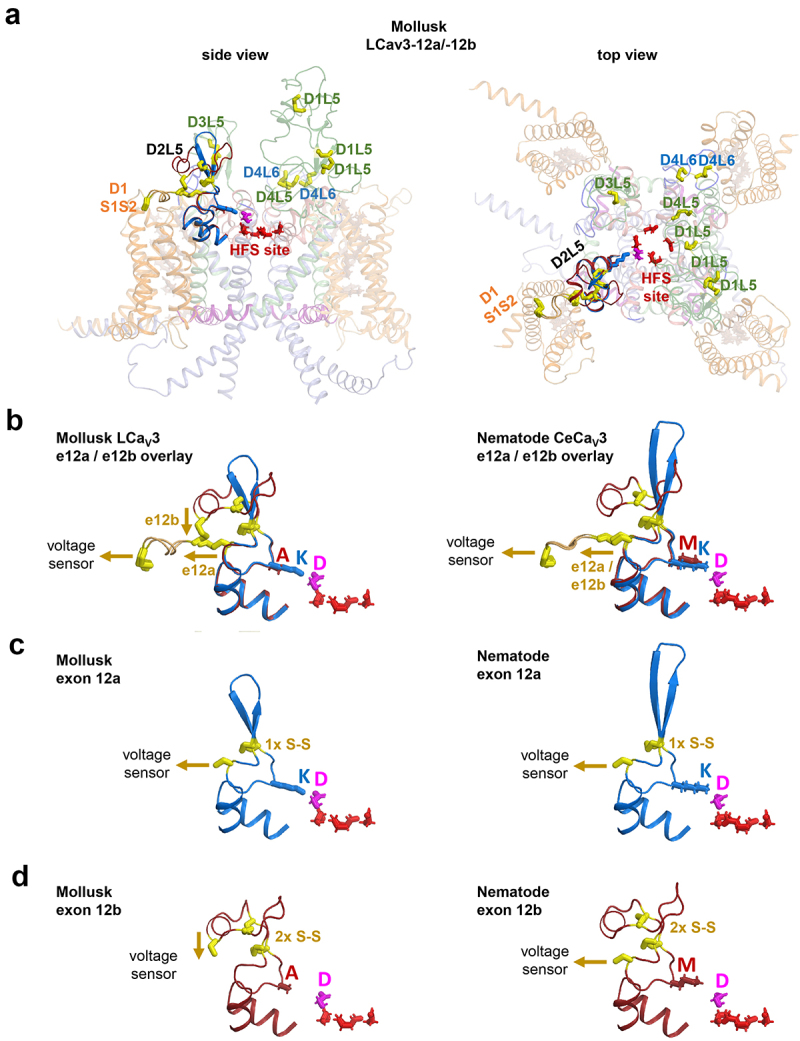
AlphaFold2 structures of molluscan and C. elegans Cav3 T-type channels with exons 12a or 12b. (A) Full-length molluscan LCav3. Highlighted are the overlay of exon 12a/12b harboring ten (exon 12a) or eleven (exon 12b) cysteine bridges in extracellular loops. (B) Overlay of exon 12a and exon 12b illustrating the differences in the location of the cysteine bridge connecting to S1-S2 extracellular loop in the voltage sensor domain D1, inside the two intra-turret cysteine bridges of exon 12b in molluscan LCa_V_3-12b but not nematode LCa_V_3-12b. (C) a lysine (K) residue approaches to neutralize the *beacon* aspartate (D) in the *beacon* (HFS^+1^ position of the Domain II outer pore in exon 12a. (B) Exon 12b has a neutral residue (A, M, S or T) in position of the lysine (K) in exon 12a of molluscan and nematode Cav3 channels.

All Ca_V_3 channels possess three to seven extra cysteine bridge pairs, most of which support the lower profile extracellular loops in L5_II_ and L5_IV_/L6_IV_ (Table 1). The distribution of the ten and eleven disulfides in representative molluscan and nematode Cav3 channels with exons 12a and 12b, respectively, are illustrated in Figure 3. A central hub of disulfides can be observed reinforcing the different folding of the short L5_II_ extracellular loop in exon 12a and exon 12b (Figure 3). A disulfide bridge from the short L5_II_ extracellular loop tethers to the voltage-sensor module by means of the S1-S2_I_ extracellular loop of Domain I, which is reinforced with an additional disulfide [59]. Differing tent pole-like restraints involving disulfides likely ensure the stabilization of the short L5_II_ extracellular loop in positioning the lysine (K) residue in exon 12a, to lie adjacent and neutralize the “*beacon*” aspartate residue in Domain II [48]. The precise positioning of the lysine to neutralize the “*beacon*” aspartate residue in Domain II, generates sodium-permeable CaV3 channels. Such positioning is not apparent in exon 12b, which contains a neutral residue (A, M, S, T) of mollusks and nematodes in position homologous to that of the lysine residue in exon 12a^42^.

The ion selectivity switch involving exon 12a and 12b is observed to require intact disulfides within L5_II_ extracellular loops, with a dramatically altered P-loop folding in response to disruption by cysteine-to-alanine mutation in molluscan Ca_V_3 channels. The altered folding results in a dramatic change in the relative Na^+^ permeability, a changing sensitivity to external Ca^2+^ block of the Na^+^ current, a changing permeability of divalent ions of barium (Ba^2+^) or strontium (Sr^2+^) compared to Ca^2+^, and a change in potency of Zn^2+^ and Ni^2+^ block, including a change in drug blocking phenotype in the loss of the typical slowing of inactivation kinetics during Zn^2+^ block after cysteine mutations in the L5_II_ extracellular loop [49].

**Figure 4.**
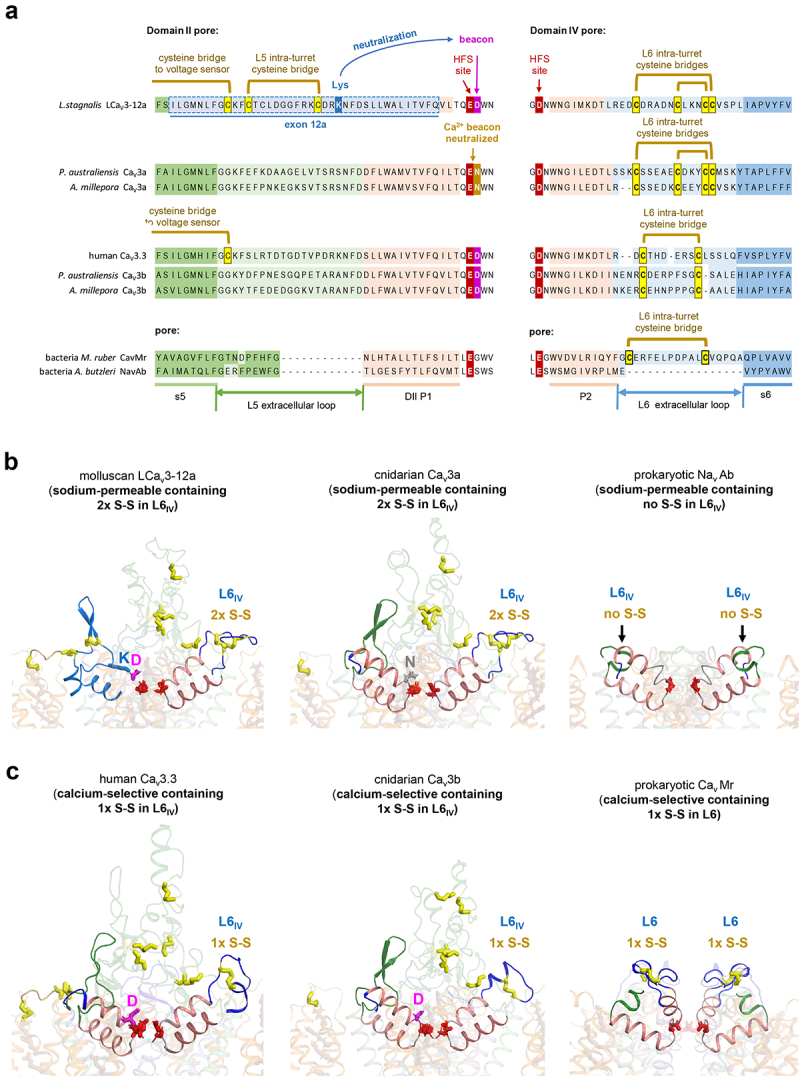
Calcium-selective and sodium-permeable channels vary systematically in the number of disulfide bridges in L6 extracellular loops located at a distance from the selectivity filter. (A) Amino acid alignments and (B, C) Alpha Fold2 (DeepMind, Alphabet), structural models of regions of representative Ca_V_3 channels spanning the ion selective pore of Domain II (including S5 segment, L5 extracellular loop, P1 helix) and Domain IV (including P1 helix, P2 helix, L6 extracellular loop and S6 segment). Equivalent regions of the subunit of homomultimeric bacterial Na_V_Ab and Ca_V_Mr channels are illustrated too. Anthozoan cnidarians possess one gene resembling the calcium-selective Ca_V_3 channels with an outer pore, *beacon* aspartate (D) in Domain II and a separate gene is configured to generate more sodium-permeant Ca_V_3 channels where a neutral asparagine residue (N) replaces the *beacon* aspartate (D) residue. Molluscan LCa_V_3-12a channels are sodium permeant because the lysine (K) residue in II_L5_ extracellular loop approaches and neutralizes the beacon aspartate (D) in the selectivity filter of Domain II. The two Ca_V_3 genes from representative anthozoan cnidarians (*Porites australiensis* and *Acropora millepora*) are illustrated. Cnidarian “Ca_V_3a” isoform resembling invertebrate sodium permeant isoforms, such as molluscan LCa_V_3-12a with a neutralized *beacon* aspartate residue in Domain II, and two set of cysteine bridges in the DIV L6 extracellular loops. (C) a cnidarian “Ca_V_3b” isoform resembling more calcium-selective human Ca_V_3 isoforms, such as Ca_V_3.3 possessing a *beacon* aspartate residue in Domain II outer pore and only one cysteine bridge in the DIV L6 extracellular loop. Bacterial calcium channel Ca_V_Mr shares a selectivity filter resembling bacterial sodium channel, Na_V_Ab, but strikingly varies in possessing an L6 extracellular loop extension containing a disulfide bridge like animal calcium channels in Domain IV L6 extracellular loop.

Importance of the “*beacon*” position associated with a configuration of disulfides within extracellular loops for generating sodium-permeable Cav3 channels were confirmed by analyzing different invertebrates. Anthozoan cnidarians possess the only known Ca_V_1, Ca_V_2 or Ca_V_3 channel gene to date in animals, which lack a “*beacon*” aspartate in Domain II [48]. These anthozoan cnidarians are the only non-vertebrates outside of flatworms to possess two genes, instead of one Ca_V_3 channel gene [48]. Presence of two cnidarian Ca_V_3 genes appears to serve an equivalent role as alternatively spliced exons 12a and 12b in mollusks and nematodes (Figure 4). Cnidarian Ca_V_3a channel is akin to sodium-permeable Ca_V_3 channels with the lysine within alternatively spliced exon 12a neutralizing the *beacon* aspartate, but in cnidarian Ca_V_3a the *beacon* aspartate is replaced by an asparagine (Figure 4a). In contrast, cnidarian Ca_V_3b appears to be equivalent to calcium-selective molluscan Ca_V_3 containing alternatively spliced exon 12b with an unmodified *beacon* aspartate.

An obligatory feature for generating the sodium-permeable T-type channels of molluscan Ca_V_3-12a and the cnidarian Ca_V_3a are the presence of two pairs of disulfides in the IV_L6_ extracellular loop (Figure 4a,b) instead of one pair of disulfides in IV_L6_, observed in calcium-selective Ca_V_3 channels of mammals (Ca_V_3.1, Ca_V_3.2 and Ca_V_3.3) and cnidarian Ca_V_3a (Figure 4a–c) [49]. Sodium permeability in a mammalian Ca_V_3.2 channel background (normally calcium-selective) requires the transfer of the IV_L6_ extracellular loop from molluscan LCa_V_3 in addition to the exon 12a containing II_L5_ extracellular loop from molluscan LCa_V_3 [49]. And in reverse, the high calcium selectivity of mammalian Ca_V_3.2 channel was only possible to attain after the transference of both II_L5_ and IV_L6_ extracellular loops onto the molluscan LCa_V_3 background.

In a similar manner, the ion selectivity may be altered in one-domain bacterial channels with differences in disulfide bridge content of the L6 extracellular loop. Bacterial sodium channels (e.g. Na_V_Ab and Nachbac) classically lack residues composing the long L5 or L6 extracellular loops observed in the eukaryotes (see Figure 1). Ca_V_Mr, a bacterial calcium channel, contain a striking extension to its L6 extracellular loop containing a disulfide bridge, resembling the animal Ca_V_3 channels with a variable configuration of disulfide-containing IV_L6_ extracellular loops which influences the sodium and calcium selectivity (Figure 4a–c).

A differing configuration of disulfides harbored in II_L5_ extracellular loop in Ca_V_3 channels enables exon 12a to directly engages the pore selectivity filter with a precisely positioned lysine residue to neutralize the “*beacon*” aspartate. Additional disulfide bridges in the IV_L6_ extracellular loop are harder to reconcile in inducing changes in ion selectivity changes, since the IV_L6_ extracellular loop is positioned too far from the pore selectivity filter to have a direct influence on it. Disulfide bridges in L6 extracellular loops likely have supportive role, acting as restraining straps to limit dynamic motions of the selectivity filter, rather than directly influence ion selectivity, *per se*.

## Different cysteine tethering to the voltage-sensor module in exon 12a generates a kinetically slower T-type sodium channel appropriate for the molluscan heart

Available molluscan and nematodes sequences, including model organism, *C. elegans*, possess a lysine residue in the L5_II_ extracellular loop adjacent to the “*beacon*” aspartate, to generate a mostly Na^+^-passing T-type channel with exon 12a, whereas a neutral residue (alanine, methionine, serine, or threonine) in identical position to the lysine residue generates a mostly Ca^2+^ permeating T-type channel with exon 12b [48].

**Figure 5.**
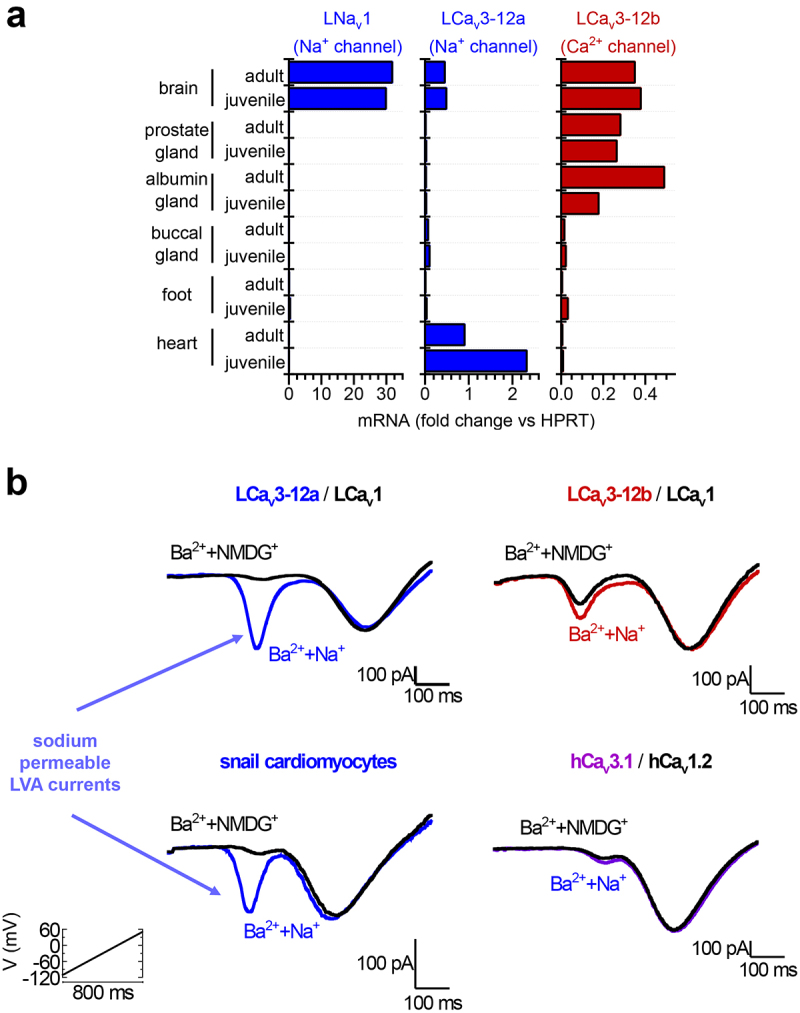
Sodium-permeable Cav3 T-type channel splice isoform with exon 12a expressed in the heart *in lieu* of LNa_V_1 sodium channel which does not express outside the molluscan nervous system. (A) mRNA transcript expression measured as fold change to control molluscan HPRT illustrate that LNav1 sodium channels only express in the nervous system, with a sodium-permeable LCav3 T-type channel with exon 12a expressed in the heart. (B) Voltage ramp currents of snail cardiomyocytes resemble those in HEK-293T cells co-transfected with sodium-permeable and mibefradil-sensitive low voltage-activated (LVA) currents from LCav3-12a T-type channel gene and sodium-impermeant, nifedipine-sensitive, high voltage-activated (HVA) currents from LCav1 L-type channel gene. Adapted from Fig. 6, Senatore *et al*. (2014) The Journal of Biological Chemistry 289:17:11952 –11,969 [54].

Mollusks, like other invertebrates outside of nematodes, possess a traditionally fast, Na^+^-selective Na_V_1 channel, but its expression is limited to the nervous system (Figure 5a) [54]. The Na^+^-permeant T-type channel with exon 12a is the only isoform of T-type channel expressed in the snail heart where it serves as a surrogate Na^+^ current in the absence of expression of an Na_V_1 channel (Figure 5a) [54]. As illustrated in Figure 5b, a voltage ramp protocol applied to isolated patch clamped snail cardiomyocytes reveals a low-voltage activated, mibefradil- and nickel-sensitive T-type sodium current, and a high-voltage activated, nifedipine-sensitive, L-type calcium current [54]. This configuration of a T-type sodium current and L-type calcium current in snail cardiomyocytes can be replicated in voltage ramp experiments involving the heterologous co-expression of sodium-permeant LCa_v_3 with exon 12a and LCa_V_1 from snails in human HEK-293T cell lines (Figure 5b) [54].

A notable structural difference in sodium-permeant LCa_V_3 with exon 12a, is that the cysteine bridging the voltage sensing module is within rather than outside the two pairs of intra-turret cysteines (Figures 1, 3) [48]. This differing location of the cysteine tethered to the voltage sensor domain for exon 12a, appears to reflect the requirements of kinetically slow, sodium-permeable Ca_V_3 T-type channels within the molluscan cardiovascular system. Exon 12a imparts a dramatically slowing of its activation, inactivation, deactivation, and recovery from inactivation in LCa_V_3 T-type channels compared to exon 12b [54]. A slowing of kinetics of LCa_V_3-12a suits the requirements of a surrogate Na^+^ current in the snail heart, just like the vertebrate heart specific Na_V_1.5 gene in vertebrates [60], to establish a cardiac action potential with a prolonged repolarization phase required for refilling of the heart, with a built-in refractoriness in the sodium current to prevent ectopic heart beats. While a co-opting of a kinetically slower, Na^+^-permeant T-type channel serves the purposes of mollusks *in lieu* of expression of their Na_V_1 channel gene within molluscan hearts, *C. elegans* lack expression of any Na_V_2 or Na_V_1 sodium channel gene. Nematode Ca_V_3 channels possess a cysteine linkage to the voltage sensor from the L5_II_ extracellular loop that is always outside the one and two intra-turret cysteine bridges for both exons 12a and exon 12b. This configuration of exon 12a and exon 12b within nematodes provide an optional sodium permeability for the nematode Ca_V_3 channel to enable a faster action potential spike generation, such as in the pharynx of *C. elegans*, where voltage-gated sodium currents have been observed [61,62].

## Differences in the expression and selectivity of calcium and sodium channel pores

The selectivity of the pores for Na^+^ and Ca^2+^ ions are not absolute or equivalent in sodium-selective and calcium-selective channels. Traditional animal Ca_V_1 and Ca_V_2 channels possess high Ca^2+^ selective over Na^+^ (~1,000 to 1) on the condition that external Ca^2+^ is present at low micromolar levels [63–67]. A tight regulation of selective Ca^2+^ entry is of salient importance to cells as a key messenger of local, intracellular Ca^2+^ signaling triggering a cascade of different functions within cells and to their intracellular compartments [68]. Most intracellular Ca^2+^ ions are not free but are bound to a myriad of different intracellular Ca^2+^ handling proteins, in affinities which differ within cells, and from cell to cell [68]. Requirement for the titration of microtargeted doses of Ca^2+^ influx, at precise time and space along the plasma membrane, necessitates a high Ca^2+^ selectivity within Ca_V_1 and Ca_V_2 channels, and less so for Ca_V_3 channels [49,69] which have a more electrogenic, pace-making function in the nervous and cardiovascular systems. Na^+^ is relatively inert in comparison to Ca^2+^ and tolerated in much greater abundance outside and inside of cells (in seawater, freshwater and extracellular fluid). It contributes to a fifteen to fifty times faster transmembrane flux rate than Ca^2+^ or K^+^ ions, at > 10 million ions per second per channel, required for generation of rapid action potential spikes [70,71]. While the Na^+^ flux rate can be exceptionally high, traditional Na_V_1 channel pores are ~ a hundred times less selective than a Ca_V_1 or Ca_V_2 pore, where ~one Ca^2+^ ion typically enters for every ten Na^+^ ions [70,71]. Allowances for measurable Ca^2+^ permeating through sodium pores is likely a feature, not a defect in Na_V_1 channels given that a monitoring of Ca^2+^ flux with bound calcium sensor, calmodulin (CaM) applies generally to sodium-selective pores rather than to calcium-selective pores [72]. The Ca^2+^ flux through Na_V_ channels may contribute to shortening action potential repolarization by co-activation of large-conductance BKCa channels [73]. The rise in localized Ca^2+^ through Na_V_1 channels is expected to be especially acute increasing to micromolar concentrations where Na_V_1 channels are clustered such as in nodes of Ranvier and axon initial segments [73]. All animal four-domain channels (Ca_V_1, Ca_V_2, Na_V_1, Na_V_2, NALCN), and likely more than half of the Ca_V_-like channels representatives in life forms outside of animals possess a conserved proximal C-terminus spanning from the end of S6_IV_ to a putative calcium sensor, calmodulin bound to the “IQ” region.

## A chemical inventory of prokaryotic and eukaryotic sequences reveals four classes of selectivity filters governing sodium permeability and calcium selectivity

**Figure 6.**
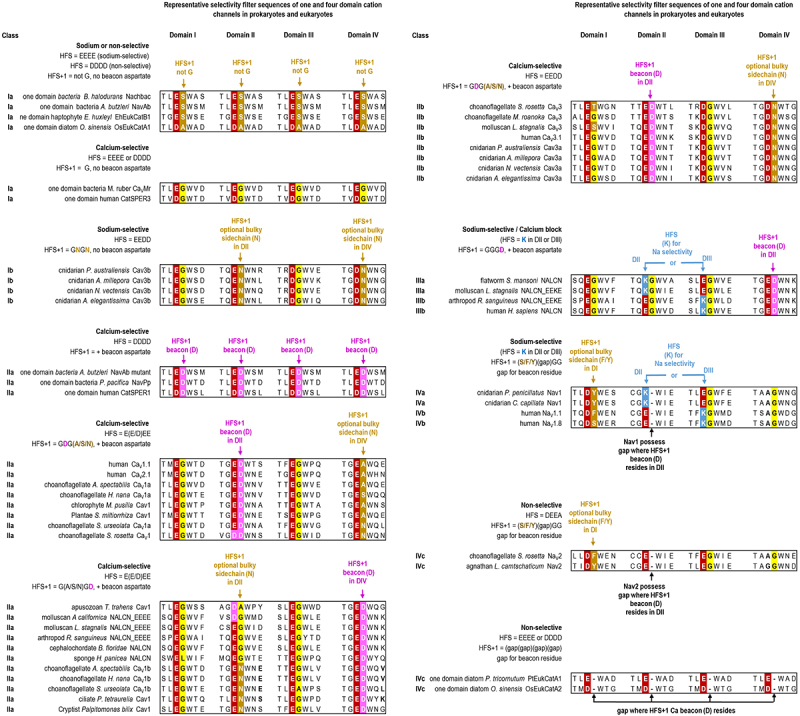
Representative selectivity filter sequences of one- and four-domain cation channels in prokaryotes and eukaryotes can be categorized into four major classes. Class I selectivity filters represent sodium-selective channels lacking a beacon aspartate in the High Field Strength (HFS) +1 position. These Class I channels include (Class Ia) bacterial sodium channels and (Class Ib) unique Cav3b T-type channel isoform found in cnidarians. Class II selectivity filters possess a beacon aspartate in the (HFS^+1^ position which in animal Cav1 and Cav2 channels is in Domain II but residing in Domain IV in some non-animal Cav channels and NALCN. Cav3 T-type channels always possess a beacon aspartate in HFS^+1^ of Domain II (Class IIb), except in a unique Cav3b T-type channels in cnidarians (Class Ib). Class III selectivity filters possess a beacon aspartate in the HFS^+1^ position in Domain IV, but also contain a HFS site with a lysine in Domain II (Class IIIa) or Domain III (Class IIIb). Class IV selectivity filters possess a gap in position of the beacon aspartate in Domain II in multiple aligned sequences of animal Na_V_2 and Na_V_1 sodium channels. Class IV selectivity filters can possess a lysine in the HFS site in Domain II (Class IVa) or Domain III (Class IVb) or lack a lysine residue in Domain II or III of the HFS site (Class IVc).

**Figure 7.**
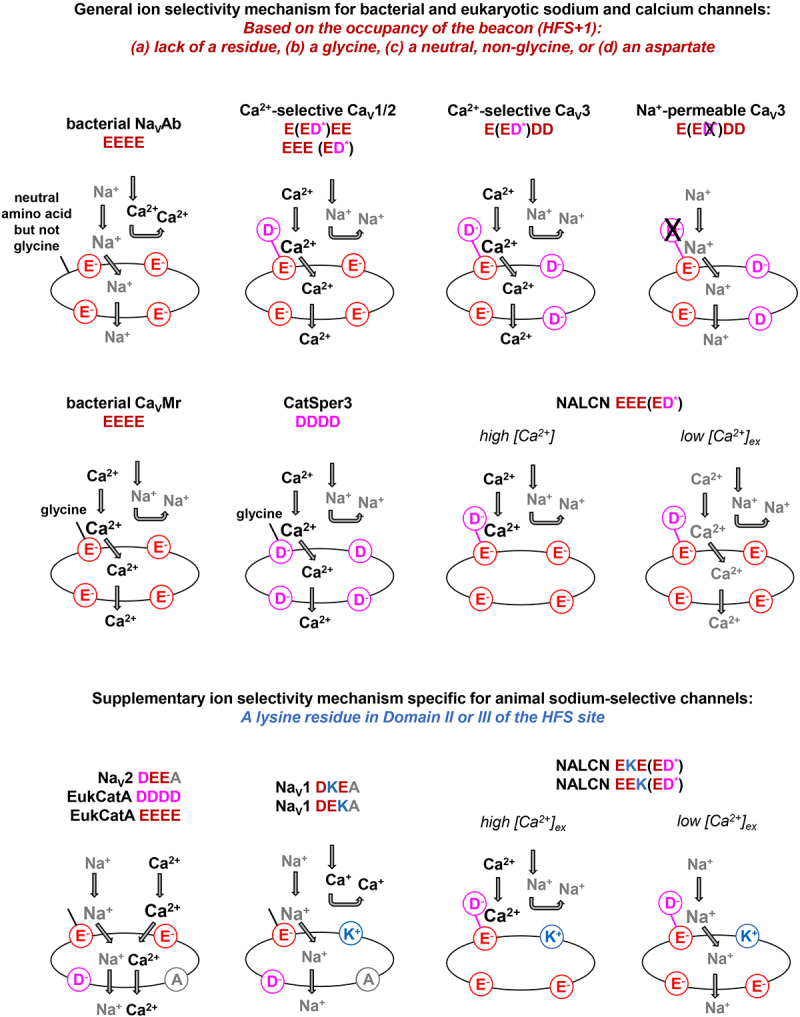
Model for the preference of Ca^2+^ or Na^+^ ion selectivity through one- and four-domain channel pores, in bacterial and eukaryotes. A key determinant for ion selectivity in the selectivity filter is the occupancy of the *beacon* High Field Strength +1 (HFS^+1^) site, lying just above the constricted, minimal pore radius of the electronegative ring of the HFS site. If the *beacon* site is a gap or glycine, it is more calcium selective, but if a neutral, small amino acid, it is more sodium permeable. The scale of permeabilities is set by the HFS which could be negatively charged amino acids aspartate (D) or glutamate (E), or in animal Na_V_1 or NALCN channels only, positively charged lysine (K). If the beacon is occupied by an aspartate residue, it is a calcium-selective channel or an ion channel with a sodium conductance blocked by physiological (mM) concentrations of external calcium.

Analyses of known sequences containing calcium channel-like and sodium channel-like pores (gathered from published literature, supplemented with BLAST search analyses of Transcriptome Shotgun Assembly (TSA) and other NCBI nucleotide databases) reveal a distinct pattern of conservation coding for the High Field Strength (HFS) site and HFS^+1^ *beacon* site of the selectivity filter. Almost all calcium and sodium pores fall into one of four distinct classes of selectivity filters based on the occupancy of the *beacon*. Representative selectivity filter sequences of each class are illustrated in Figure 6, a summary of the classification scheme of ion selectivity based on occupancy of the *beacon* is provided in Table 2 and cartoon models of the occupancy of High Field Strength (HFS) site and corresponding preference of calcium or sodium ion selectivity through one- and four-domain channel pores is illustrated in Figure 7.

**Table 2.** Summary of a proposed classification of selectivity filters of one- and four-domain voltage-dependent sodium, calcium and NALCN channels in prokaryotes and eukaryotes.

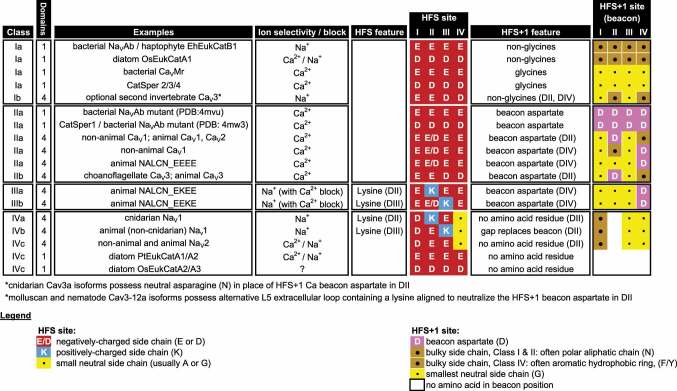

Class I selectivity filters represent those lacking a *beacon* aspartate in the High Field Strength (HFS) +1 position. These include bacterial and eukaryotic one-domain (monomeric subunit) channels (Class Ia), and a unique, four-domain and sodium-permeable Cav3b T-type channel found in cnidarians (Class Ib) [48]. Even though the *beacon* facilitates calcium selectivity when occupied by an aspartate residue, there appears to be a correlation of ion selectivity phenotypes associated with the occupancy in the beacon HFS^+1^ position.

When the beacon of Class Ia selectivity filters are occupied by the smallest amino acid, glycine, the one-domain channels are Ca^2+^ selective, regardless of whether the HFS position is occupied by glutamate (e.g. sperm calcium channel, CatSper2/3/4 [74] or aspartate (e.g. bacterial Ca_V_Mr [75].

When the beacon position is occupied by a a one-domain channel, it is a sodium-permeable channel. In this case, sodium selectivity is highest when the HFS position is occupied by glutamate (e.g. bacterial Na_V_Ab [34] or NachBac [45] or haptophyte EhEukCatB1 [76] or dinoflagellate EukCatC [76], and is nonselective for sodium and calcium ions when the HFS site is occupied by aspartate (e.g. marine diatom OsEukCatA1 [77].

Class II selectivity belong to calcium-selective channels that contain a *beacon* aspartate, which can occupy all four domains (e.g. in the one-domain sperm calcium channel, CatSper1 [78] or be restricted to Domain II only (in animal four-domain Ca_V_1 and Ca_V_2 channels [29] or be restricted to Domain IV only (in four-domain NALCN channels with calcium-like pores [12] and many non-animal Ca_V_ channels).

Four-domain Ca_V_3 T-type channels possess a *beacon* aspartate in Domain II (Class IIb), except when it is lacking in the alternative Cav3b T-type channel isoform in cnidarians (Class Ib) [48]. The HFS position in Ca_V_1, Ca_V_2 and NALCN channels from Domain I to Domain IV is always EEEE or, more rarely, EDEE [29]. The equivalent HFS position of Ca_V_3 channels (Class Ib/IIb) is always EEDD, from choanoflagellate to human T-type channels [48].

Class III selectivity filters are animal NALCN homologs possessing a *beacon* aspartate in Domain IV like Class II selectivity filters of calcium-selective channels found in some non-animal species [48]. Class III selectivity filters are distinguished from Class II selectivity filters in containing a HFS site with a lysine in Domain II (Class IIIa) or Domain III (Class IIIb) [12]. The combination of the HFS of Class III selectivity filters resembling Na_V_1 channels with a lysine in Domain II or III and the presence of the *beacon* aspartate in Domain IV resembling calcium channels appears to contribute to NALCN’s dual calcium/sodium neutral amino acid other than glycine in phenotype: a sodium leak current of tiny single channel conductance, with a high affinity block with external calcium at physiological (mM) concentrations [33,50,52]. Animal NALCN channels are always restricted to the smallest, neutral amino acid, glycine in beacon positions (Domains I, II and III) that is not the beacon aspartate residue (Domain IV).

Class IV selectivity filters contain the animal, sodium (Na_V_2 and Na_V_1) channels (Class IVa/IVb), and these selectivity filters always possess a lack of a residue in the multiple sequence alignment compared to where the *beacon* aspartate is positioned to promote calcium selectivity in animal Ca_V_1, Ca_V_2 and Ca_V_3 channels [79]. The apparent “gap” in position of the *beacon* aspartate in the multiple sequence alignment is not visible as a defined “space” *per se* in homologous position of the beacon position in three-dimensional structure. The gap is spread across the selectivity filter in sodium channels, which is shortened by one amino acid compared to the homologous selectivity filter of calcium-selective channels in Domain II. The highest sodium selectivity is defined by the presence of a lysine in the HFS site in Domain II (Class IVa) [39] or Domain III (Class IVb) [37] of animal Na_V_1 channels, where the lack of a lack of a lysine residue in Domain II or III of the HFS site generates Na_V_2 channels, nonselective for sodium and calcium ions, (Class IVc) [40]. Many, but not all, Na_V_2 or Na_V_1 channels, defy the ubiquity of a glycine in the beacon position of Domains I and III of four-domain channels, with a possible aromatic hydrophobic ring (F or Y) in the beacon position of Domain I. One-domain EukCatA channels found in diatoms possess Class IVc selectivity filters also. EukCatA channels appear to be the one-domain version of the four-domain animal Na_V_2 channels, in possessing of a lack of a residue in position of the beacon in multiple sequence alignments and also lacking a lysine residue in the HFS [77]. One-domain PtEukCatA1 channels are nonselective for sodium and calcium ions, just like their four-domain counterparts, in animal Na_V_2 channels [77].

One- and four-domain calcium or sodium, or NALCN channels in bacteria and eukaryotes, possess a variable number of negative charges within the outer vestibule above the selectivity filter at position HFS^+3^ or HFS^+4^, which is most striking for Na_V_2 and Na_V_1 channels which often forms a complete outer ring of negatively charged residues, from Domain I to Domain IV, EEDD [35] (Table 2). The large number of negative-charges in the outer pore vestibule, attracts positive cations to the pore, can alter the pore blocking affinity of positively charged toxins (e.g. tetrodotoxin and saxitoxin) and divalent cations, and also alter ion conductances [80]. Neutralization of negative charges in the outer pore has been attributed to the selective pressure in animals attempting to evade the pore blockade by bacterial toxin TTX and algal toxin STX [35]. The outer ring of negative charges is much less consequential compared to the beacon site in influencing ion selectivity. The outer ring of negative charges in the HFS^+3^ and HFS^+4^ positions are farther from the beacon site which is adjacent to the minimum orifice of the HFS position in the selectivity filter.

## Consideration of pore dimensions on ion selectivity

**Figure 8.**
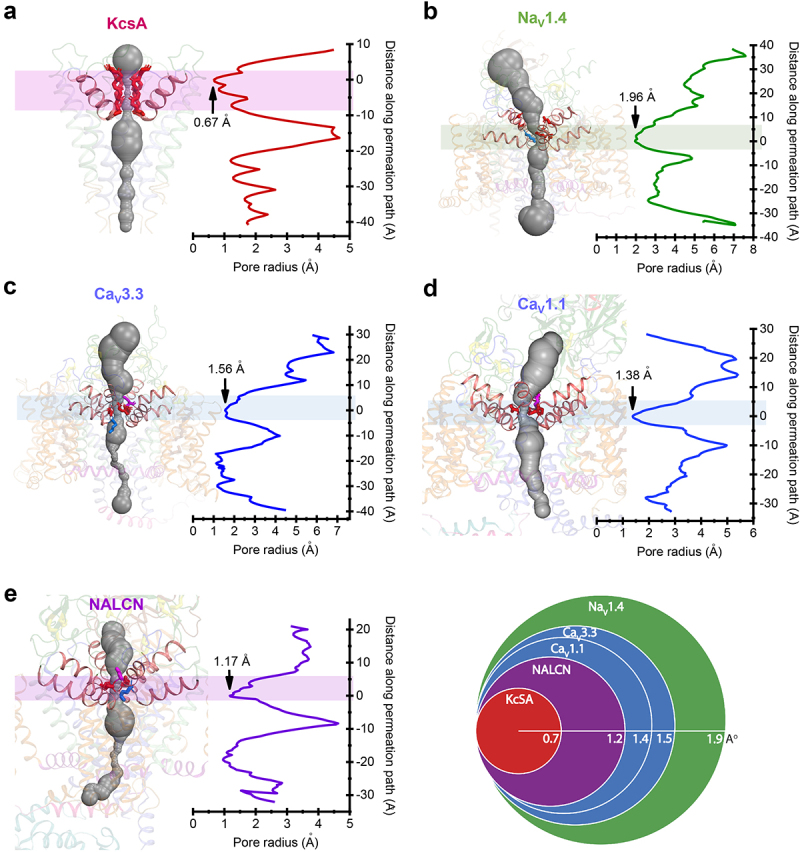
**Estimates for the pore dimensions of representative sodium, calcium, and potassium channels**. Pore dimensions for X-ray crystallographic structure for: (A) bacterial potassium channel KcsA from *Streptomyces lividans* (Pdb:1f6g) [81] and cryo-EM derived structures: (B) human Nav1.4 (PDB: 6agf) [82], (C) human Cav3.3 (Pdb:7wll) [32], (D) rabbit Cav1.1 (Pdb:5gjv) [56] and (E) human NALCN (Pdb:6×iw) [50]. Panels a to E: Space-fill (gray color) of ion channel pore dimensions (left side of panel), and graph of pore radius along the permeation path through the selectivity filter (right side of panel). Minimum pore radius indicated at end of arrow on graph, with the region containing the minimum radius of the selectivity filter highlighted by a band of color. (A) Backbone residues of TVGYGD selectivity filter residues of KcsA illustrated in red sticks. (B-E) Charged selectivity filter residues illustrated in sticks (K=blue, D/E=red, beacon aspartate=magenta); (F) Overlap of average minimum pore radius of ion selectivity filters illustrates a rank order of pore radii from largest to smallest: Na_V_1.4 >> Ca_V_3.3 > Ca_V_1.1 > NALCN >> KcsA. Ion channel pore dimensions estimated using CAVER 3.0 PyMol plugin [83] within PyMol 2.5.2 (Schrödinger, LLC). Estimates of channel pore dimensions using CAVER 3.0 were very similar to those estimated using HOLE [84], CASTp 3.1 [83], MOLE 2.5 [85], and Pore Walker [86].

The classification of selectivity filters based on the beacon residue described above illustrates that there is a consensus pattern of governance of ion selectivity across disparate channel types, from single-domain and four-domain channels, from prokaryotes, single cell eukaryotes, to the animals, and across different classes of sodium and calcium channels. These different class of ion channels possess dramatically different pore orifice sizes along the permeation pathway, though, as illustrated with representatives of the major channel classes of experimentally derived, cryo-EM channels in Figure 8. The average predicted radius of the minimum pore of the selectivity filter (illustrated in Figure 8, panels a to e) in rank order of pore radii (from largest to smallest) include: Na_V_1.4 [82] (1.96 Å) >> Ca_V_3.3 [32] (1.56 Å) > Ca_V_1.1 [56] (1.38 Å) > NALCN [50] (1.17 Å) >> KcsA [81] (0.67 Å). Estimated values of the minimum pore can vary based on assumptions of van der Waals radii [84]. Findings of minimal pore radii are close to those previously reported, except for NALCN [50], where a pore radius for NALCN is estimated to be smaller rather than larger than the minimum pore radius of Ca_V_1.1.

Regardless of minor variations in estimations, the overall minimum pore radius of selectivity filters between the different ion channels are dramatically different no matter which method you use to calculate the minimum pore radii (see Figure 8g), with differences between the largest minimal pore radius (Na_V_1.4) and the smaller minimal pore radii of Ca_V_1.1 and NALCN approximating the radius of the potassium channel selectivity filter (KcsA).

The potassium channels always form monomers of a one-domain subunit and are of a different architecture for the selectivity filter. Backbone residues of the potassium channel, coding for TVGYGD for KcsA, provide carbonyl oxygens that form an octet, to coordinate the occupancy of a completely dehydrated potassium ion, alternating with water molecules [87], positioned in single file along four possible positions of the selective pore (Figure 8a) [28]. Electrochemical gradients drive the potassium ion by a knock-off mechanism in a cycle of dehydration, high affinity occupancy, and rehydration from inside-to-outside, across the selective pore’s narrow conduit [28].

Compared to the narrow and orderly, single-file column of defined positions for potassium ions to transit through the selectivity filter, the sodium and calcium channels, possess a much wider and shorter pore (Figure 8b–f). The selectivity filter derived from analysis of the bacterial Na_V_Ab sodium channels is defined by the contribution of backbone carbonyls of the HFS^−2^ and HFS^−1^ positions, which lie adjacent to the narrowest, restricted access provided at the HFS site [34,88]. Ions traversing the selectivity filter are modeled to always be at least partially hydrated [34,88]. Molecular dynamics modeling suggest ion occupancy in the selectivity filter follows what appears to be a myriad of possible trajectories, where ions are cradled and dunked by highly, mobile side chains of charged amino acids, contributed by HFS and HFS^+1^ residues, mostly facing toward the pore lumen, in a selectivity filter environment described as a “*highly degenerate, liquid-like energy landscape*” [88]. Ion permeation is modeled as a knock-on mechanism by alternative occupancy of two and three sodium ions in the selectivity filter of the Na_V_Ab channel, each ion contributing to an ionic cluster of variable charge and spatial arrangement due to the variable disposition of ions and charged, amino acid side chains along the pore’s wide, but short selectivity filter [88].

Ion selectivity in the short and broad selectivity filter of sodium and calcium channels appears to be dependent on what is occupied in the *beacon* (HFS^+1^) site. This *beacon* appears to be critically positioned just above the minimal radius and constriction point of the electronegative ring (D^−^ or E^−^) of the HFS site.

Approaching from smallest to largest sizes occupying the beacon, an associated pattern is revealed in one-domain calcium and sodium channels: A lack of a residue in position of the *beacon* in a multiple aligned sequence which shortens the selectivity filter by one amino acid, results in a nonselective channel, permeable to sodium and calcium ions. Occupancy of the smallest amino acid, glycine in the *beacon* transforms the nonselective one-domain channel into one that is limited to the divalent, calcium ion (e.g. Ca_V_Mr [75]. A small, neutral, but non-glycine amino acid (e.g. serine, alanine) in the *beacon* site in a one-domain channels (e.g. NavAb [34], EhEukCatB1 [76] favors sodium permeability. Even when the HFS site contains an aspartate rather than glutamate, such as in the sperm channel CatSper2/3/4, it is a calcium-selective channel [74] with a *beacon* glycine residue. OsEukCatA1 with a small, non-glycine residue at the *beacon*, and an aspartate instead of glutamate in the HFS position is a nonselective but sodium-permeable channel [77]. Last, when the *beacon* is an aspartate residue, it is always a calcium-selective channel regardless of whether the HFS position is a glutamate such as in bacterial Na_V_Ab mutant (PDB:4mvu) [47] or an aspartate residue such as in CatSper1 [78].

Ion selectivity phenotype is similarly governed by the occupancy of the beacon (HFS^+1^) site in four-domain channels. Notably, the smallest amino acid, glycine occupies every position of the beacon position in all four-domain channels except:

- in calcium-selective Ca_V_1, Ca_V_2 and Ca_V_3 channels, where a negatively charged *beacon* aspartate and a neutral asparagine lie across from each other on either side of the selective pore in Domains II and IV just above the constriction point of the ring of negative-charged glutamates and aspartates of the HFS (composed of EEEE, EDEE or EEDD). The *beacon* aspartate/asparagine combination in Domains II and IV, likely provide a conceptual ionic sieving favoring calcium ion passage over sodium ions through the electronegative HFS site below the *beacon* in the selectivity filter.
- in sodium-permeable Na_V_2 and Na_V_1 channels, there is always a lack of a residue in position of the multiple sequence alignment where the *beacon* resides in Domain II, and a non-glycine residue that is often an aromatic ring (phenylalanine, F or tyrosine, Y) in Domain I. This is the configuration for four-domain sodium-permeable channels, which range from highly sodium-selective Na_V_1 channels with a lysine in Domain II or Domain III of the HFS site, and nonselective channels for calcium and sodium ions in the absence of a lysine in Domain II or Domain III of the HFS site (Na_V_2 channels).
- in NALCN channels, which appears as a hybrid of (a) and (b) above, with (a) a *beacon* aspartate in Domain IV, and (b) an optional lysine in Domain II or Domain III of the HFS site. Notably, every residue in the *beacon* site in every animal NALCN channel except for the *beacon* aspartate in Domain IV is a glycine residue.

## Evolutionary diversity of selectivity filters for calcium and sodium ions

The one-domain sodium and calcium channels in bacteria are broadly distributed, but absent in the majority of bacterial species [89]. The one-domain eukaryotic sodium and calcium channels, dubbed the “EukCats” also have a finite distribution, limited to species of Stramenopiles, Cryptophytes, Haptophytes and Alveolates [76,77] (Table 3). Likewise, one-domain CatSper is a complex of genes that are completely missing in different animal lineages, and yet also retained in whole within disparate groups of different, unrelated, single-cell eukaryotes [90] (including species of yeast, apusozoans, Stramenopiles and Glaucophytes). CatSper is a complex of genes including CatSper1/2/3/4 and CatSperβ, all required in calcium-mediated sperm motility activated by the alkaline conditions of the female reproductive tract [74]. Interestingly, one-domain CatSper appears to be functionally replaced by an unusual four domain, cyclic nucleotide gated potassium channel (CNGK) in a few animal groups, and in different single cell choanoflagellate species which can possess a CNGK homolog [91,92]. Overall, the spotty distribution of all one-domain calcium and sodium channels suggests that their diversification amongst eukaryotes is likely the result of horizontal gene transfer between different bacteria and particular eukaryotes, for fulfilling specific functional niche requirements.

**Table 3.** Diversity of one- and four-domain voltage-dependent sodium, calcium, and NALCN channels in eukaryotes.

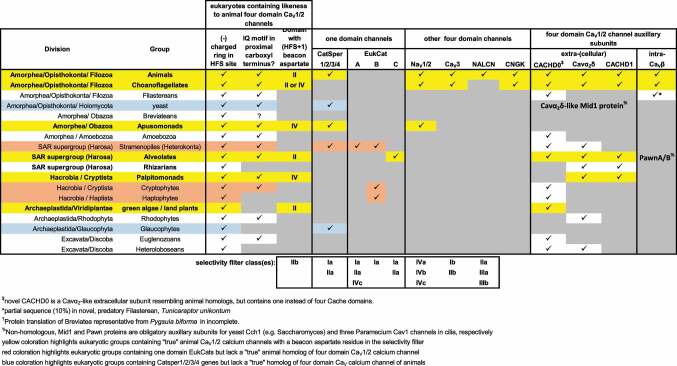

A one-domain channel ancestor likely underwent two rounds of duplication to generate a four-domain stem template that radiated into the five different animal classes [93]. Evidence that animal Na_V_2, Na_V_1, Ca_V_1, Ca_V_2, and Ca_V_3 (but not NALCN) channels have a common shared inheritance is in the conservation of their splice sites in the first of four domains, including a rare AT-AC, U12-type splice site [58]. All animal four-domain channels possess a similar asymmetry in possessing lengths of extracellular loops and cysteine bridges that are similar across these different channels [29], as well as the relative lengths of the cytoplasmic III-IV loop that occupies a crevice formed by the proximal C-terminus containing a conserved, calmodulin binding IQ motif [29] except for Ca_V_3 channels, where a universal calmodulin binding domain is located at the *gating brake* of the I-II linker [94].

All major eukaryotic groups have representative species with a distant likeness to the four-domain channels with negatively charged residues in the HFS site (Table 3). Notably, only five of seventeen of these eukaryotic groups outside of animals contain the characteristic features of the selectivity filters of animal calcium channels, including the *beacon* aspartate (in Domain II or Domain IV) and an animal HFS site that is either EDEE or EEEE [29]. A strict classification based on a presence of a *beacon* aspartate limits the group of animal-like calcium channels to include the familiar single cell eukaryotic models for studying animal calcium channel signaling, including *Paramecium* [95] (Alveolate) and *Chlamydomonas* [96] (green algae), as well as basal cryptist, *Palpitomona* [97], the apusozoan *Thecamonas*, and the closest living ancestors to the animal lineage, the choanoflagellates [98]. Of the single cell eukaryotes studied outside the choanoflagellates, calcium channels are limited to expression in the~4,000 cilia per ciliate of *Paramecium* [95] and the distant tips of the two flagella per green algae, *Chlamydomonas* [96], suggesting that the four-domain calcium channels may have arisen for the purposes of generating flagellar/ciliary movements, predating their infiltration in cell membranes in choanoflagellates and animals. For the most part, the one-domain calcium and sodium channels of the EukCats are inhabited in species which don’t contain the four-domain calcium channels with the *beacon* aspartate residue (Table 3). This suggests a more likely parallel evolution to generate action potential spikes utilizing one-domain calcium and sodium channels within marine phytoplankton (Stramenopiles), Cryptophytes and haptophytes *in lieu* of utilizing those resembling four-domain homologs in animals.

Choanoflagellates are unique compared to other single cell eukaryotes, in containing a “true” set of gene homologs of the animal calcium and sodium channel variety, including an L-type calcium channel (Ca_V_1), a T-type channel (Ca_V_3) and a sodium channel, (Na_V_2). The choanoflagellates (and likely the Filasterea) are unique among the basal eukaryotes in possessing an animal homolog of the intracellular, accessory, Ca_V_β subunit that complexes with the Ca_V_1 channel. Ca_V_β subunits are not featured in any eukaryotic group outside the Filozoa (includes: choanoflagellates, filastereans and animals). Ca_V_α_2_δ subunits are more widespread amongst non-animal eukaryotes, often possessing homologs to animal Ca_V_α_2_δ and CACHD1, as well as a novel, short Ca_V_α_2_ subunit, dubbed CACHD0, which possesses one instead of four Cache domains [99]. The lack of homologs to animal Ca_V_α_2_δ subunits in species with eukaryotic Ca_V_-like channels reduces the constraints on structure, allowing for greater variability in long extracellular loops which normally supports Ca_V_α_2_δ subunits.

Animal homologs of Ca_V_1 and Ca_V_3 calcium channel subunits are limited to choanoflagellate species and basal animals whose primary habitat is in the marine, not freshwater, environment, suggestive of a high calcium salt environment required for the transmission of meaningful calcium signals in these basal species. Na_V_2 channels are limited to choanoflagellates and apusozoans in non-animal eukaryotes and was a likely origin for the template that served in the evolution of animal Na_V_1 channels, correlating with appearances of sodium-dependent fast action potentials within the first animals bearing true nervous systems, likely in a close relative to the extant cnidarians [58]. Sodium channels have a lineage extending to the Obazoa, but while every eukaryotic and animal group contains at least one Ca_V_-like channel, sodium channels are absent in many animals, such as parasitic flatworms, nematodes, echinoderms and hemichordates. NALCN channels are the last of the calcium and sodium channels to appear in evolutionary terms. NALCN first appears in extant basal multicellular organisms such as sponge and is limited in distribution to the animals, but is found in all known animals to date, and limited to a single gene homolog in animals except for species of nematodes, cnidarians and sponges. There is a distant likeness of NALCN to Cch1 of yeast [100], but yeast Cch1 is not a NALCN homolog. Yeast Cch1 more closely resembles the divergent set of calcium-like channel structures in life forms outside of animals and choanoflagellates.

## Why did animals adopt four-domain channels (outside of sperm-specific channel, CatSper), when alternative one domain calcium- and sodium-selective channel structures are the prevalent, ancestral template from bacteria and single-cell, marine phytoplankton?

An interesting question to ponder is why animal four-domain channels have asymmetric selectivity filters with differing configuration of residues in the beacon and HFS sites of all four domains when calcium- and sodium-selective channels can simply be generated in one-domain versions in bacteria and single-cell eukaryotes. The answer likely lies in the greater spectrum of possible variations in ion selectivity requirements for different biological relevant, monovalent and divalent cations, as well as variations in selectivity filter sequences that can be used to evade blockade from animal or bacterial toxins in asymmetrical pores. An example addressing the former, is consideration of the limitations of ion selectivity possibilities in the switch of occupancy of the beacon (HFS^+1^) site in one-domain channels, which leads to nonselective channels if there is a lack of a residue in the beacon position in a multiple sequence alignment. These one domain channels are calcium-selective channel (if the beacon is a glycine or aspartate) and sodium-permeable channel (if the beacon is a non-glycine, non-aspartate residue). Unlike the one-domain channels, a lack of a residue in the beacon position of Domain II or Domain IV isn’t limited to nonselective sodium channels in animals. Differing degrees of sodium vs calcium permeability is further afforded in the occupancy of a lysine in the HFS site of Domain II or Domain III. If there is an aspartate occupying the beacon position of Domains II or IV, a highly calcium-selective channel can be generated. Alternatively, a calcium/sodium channel hybrid can be generated such as in NALCN channels, with a sodium leak current contributed in part by the occupancy of a lysine in the HFS site of Domain II or Domain III, and a strong calcium block is contributed by a beacon aspartate in Domain IV. Another major difference in the four-domain channels is the complex scaffolding available in their more extensive extracellular loops, that permits adornment of different extracellular accessory subunits (e.g. Cavα_2_, Na_V_β, NALF), which can shield and shape localized ionic environments above channel pores, before reaching the ionic permeation pathway below. These asymmetrical, extracellular loops of variable length and number of intra-turret disulfides likely allow for differing motions in each of the selectivity filters of the four domains, contributing to nuances in the activation/C-type inactivation states contributed by each of the four selectivity filters in different animal sodium and calcium channels.

## Data Availability

The data that support the findings of this study are available from the corresponding author, JDS, upon reasonable request.
